# Primary lateral sclerosis: consensus diagnostic criteria

**DOI:** 10.1136/jnnp-2019-322541

**Published:** 2020-02-06

**Authors:** Martin R Turner, Richard J Barohn, Philippe Corcia, John K Fink, Matthew B Harms, Matthew C Kiernan, John Ravits, Vincenzo Silani, Zachary Simmons, Jeffrey Statland, Leonard H van den Berg, Delegates of the 2nd International PLS Conference, Hiroshi Mitsumoto, Delegates of the 2nd International PLS Conference, Senda Ajroud-Driss

**Affiliations:** 1 Nuffield Department of Clinical Neurosciences, Oxford University, Oxford, UK; 2 Department of Neurology, The University of Kansas Medical Center, Kansas City, Kansas, USA; 3 ALS Centre, Department of Neurology, CHRU Bretonneau, Tours, France; 4 Neurology, University of Michigan, Ann Arbor, Michigan, USA; 5 Neurology, Columbia University College of Physicians and Surgeons, New York City, New York, USA; 6 Bushell Chair of Neurology, Brain and Mind Centre, University of Sydney, Sydney, New South Wales, Australia; 7 Neurology, Royal Prince Alfred Hospital, Camperdown, New South Wales, Australia; 8 Neurosciences, University of California San Diego, La Jolla, California, USA; 9 Department of Neurology & Laboratory of Neuroscience, Istituto Auxologico Italiano IRCCS, Milano, Italy; 10 Department of Pathophysiology & Transplantation, "Dino Ferrari" Center, Università degli Studi di Milano, Milano, Italy; 11 Neurology, Penn State Health Milton S Hershey Medical Center, Hershey, Pennsylvania, USA; 12 Neurology, University Medical Center Utrecht, Utrecht, The Netherlands

## Abstract

Primary lateral sclerosis (PLS) is a neurodegenerative disorder of the adult motor system. Characterised by a slowly progressive upper motor neuron syndrome, the diagnosis is clinical, after exclusion of structural, neurodegenerative and metabolic mimics. Differentiation of PLS from upper motor neuron-predominant forms of amyotrophic lateral sclerosis remains a significant challenge in the early symptomatic phase of both disorders, with ongoing debate as to whether they form a clinical and histopathological continuum. Current diagnostic criteria for PLS may be a barrier to therapeutic development, requiring long delays between symptom onset and formal diagnosis. While new technologies sensitive to both upper and lower motor neuron involvement may ultimately resolve controversies in the diagnosis of PLS, we present updated consensus diagnostic criteria with the aim of reducing diagnostic delay, optimising therapeutic trial design and catalysing the development of disease-modifying therapy.

## Introduction

Primary lateral sclerosis (PLS) is a characteristically slowly progressive and selective neurodegenerative disorder primarily affecting the adult central motor system. Progressive muscle stiffness leads to an insidious loss of mobility typically with the development of corticobulbar dysfunction, which may be the initial symptom for a minority. Diagnostic criteria for PLS proposed 75 years ago recognised the potential for clinical overlap in the early symptomatic phase with the more common disorder amyotrophic lateral sclerosis (ALS).^1^ Like PLS, upper motor neuron (UMN)-predominant ALS has a significantly slower rate of progression compared with classical forms of ALS, with survival frequently extending into a second decade from onset of symptoms.^2^ The development of clinically obvious and functionally significant, progressive lower motor neuron (LMN) involvement is inevitable in ALS, in contrast to PLS, but may not emerge for several years from the initial clinical UMN syndrome.^3^ As a result, criteria for the definite diagnosis of PLS have enshrined a minimum duration of symptoms, varying from 3 to 5 years.^1 4 5^

Among the earliest reported cases, many of those that were said to have a hereditary component^1^ would now be recognised within the spectrum of hereditary spastic paraplegia (HSP). The development of non-invasive neuroimaging has brought further structural, inflammatory and metabolic mimic disorders into consideration (see later). A ‘gold standard’ *postmortem* histopathological signature for PLS has proved elusive. While neuronal and glial cytoplasmic inclusions of the 43 kDa transactive response DNA-binding protein, TDP-43, are common to 97% of cases of ALS (across disparate monogenetic and apparently sporadic cases), there have been very few *postmortem* studies of PLS in the modern era of immunohistochemistry.^6 7^ Debate as to whether PLS represents an extreme end of a continuum with ALS, or a distinct disorder is ongoing.

The clinical imprecision in the diagnosis, along with some uncertainty about overlap with UMN-predominant ALS has become an obstacle to therapeutic development for PLS. As the result of a meeting of international PLS experts (3 May 2019, Philadelphia, Pennsylvania, USA), a working group set forth to create more permissive diagnostic criteria, in an effort to spur therapeutic development and to accelerate research into the basic histopathology of PLS.

### The core clinical syndrome

There have been consistent clinical observations reported across multiple case series in PLS.^8^ Mean age at symptom onset is around 50 years which is at least a decade earlier than non-familial ALS, and a decade later than HSP. While there have been cases reported with symptoms beginning in childhood, many of those might now be linked to developmental or monogenetically mediated disorders. A male predominance has been consistently noted in PLS (range 2–4:1).

An insidious onset is the rule in PLS, so that individuals are unlikely to reach specialised neurological services soon after the very earliest symptoms. For the majority of patients, symptoms emerge in the lower limbs first, but for a significant minority in the corticobulbar pathways with dysarthria and often prominent emotionality (pseudobulbar affect). Although dysphagia may become marked, the value of gastrostomy is far less clear than in ALS, and the need for non-invasive ventilation in PLS more exceptional. Lower limb involvement in the early symptomatic phase may be articulated as a sense of dysequilibrium or loss of fluidity in gait. Prominent sensory involvement should not be evident. Spasticity with pathological hyperreflexia are invariable examination findings. Although PLS typically generalises to include the upper limbs, a focal upper limb onset to symptoms is very unusual in PLS.^9^

### Additional clinical features

Bladder instability resulting in frequency and varying degrees of retention is a common accompaniment to PLS. The presence of wider brain involvement in PLS is increasingly recognised clinically but is typically minor in relation to the core clinical syndrome. Extrapyramidal features are occasionally observed.^10 11^ Although significant cognitive impairment within the spectrum of frontotemporal dementia (FTD) has been documented,^3 12–14^ the frequency of FTD is significantly lower than in ALS. While the initial lower limb symptom onset in PLS is commonly symmetrical, a progressive hemiplegia is a very rare phenotype originally described eponymously by Mills.^15^ Such cases need a particularly careful search for focal lesions as they may mimic ‘solitary sclerosis’.^16^ Once these have been excluded, cases of slowly progressive hemiplegia, typically with bilateral UMN signs despite unilateral weakness,^17^ are currently reasonable to consider within the spectrum of PLS.

### Consensus diagnostic criteria

A revised framework is proposed to facilitate the earlier diagnosis of PLS (box 1). The choice of a minimum age of 25 years at point of diagnosis (‘probable’ or ‘definite’ PLS) reflects a pragmatic decision to minimise the risk of highly atypical cases distorting outcome in future therapeutic trials.

Box 1 **Consensus diagnostic criteria for primary lateral sclerosis (PLS)**

The diagnosis of PLS requires:

- the *presence* of:
  - age ≥ 25 years;
  - symptoms of progressive upper motor neuron (UMN) dysfunction for at least 2 years;
  - signs of UMN dysfunction* in at least two of three regions: lower extremity, upper extremity, bulbar.
- the *absence* of:
  - sensory symptoms (unexplained by comorbid condition);
  - active lower motor neuron (LMN) degeneration†;
  - alternative diagnosis‡: UMN pathology demonstrated on neuroimaging, or identified through biofluid testing that provides a plausible alternative explanation for the clinical syndrome.

**2. Diagnostic certainty**

- *Probable PLS* is defined by the absence of significant active LMN degeneration 2–4 years from symptom onset.
- *Definite PLS* is defined by the absence of significant active LMN degeneration 4 or more years from symptom onset.

*Clinical signs, including spasticity and associated weakness, pathological hyperreflexia (including Hoffman’s sign and bilateral extensor toe responses), pseudobulbar affect. Laboratory evidence of UMN dysfunction from emerging neuroimaging, neurophysiological and neurochemical biomarkers (see ‘Emerging technology’ section) is pending validation.†Minimally increased insertional activity and positive sharp waves or fibrillation potentials in extremity muscles are permitted (see ‘Electromyographic considerations’ section).‡see ‘Differential diagnosis’ section.box 2

### Electromyographic considerations

The late development of LMN involvement in some cases of UMN-predominant ALS has the potential to lead to misclassification of PLS. This issue is complicated by the presence of ‘low-grade’, non-progressive electromyographic (EMG) signs of limited muscle denervation in some cases of PLS.^4 18–23^

Pringle *et al* allowed ‘at most, *occasional* fibrillation and increased insertional activity in a few muscles (late and minor)’.^4^ Gordon *e* *t al* divided patients into pure PLS and UMN-dominant ALS, with 13 of 29 patients with pure UMN developing EMG denervation and LMN signs on average between 3 and 4 years from symptom onset, four of who met criteria for ALS.^5^ Other series have not shown such a definitive transition to UMN-predominant ALS, nor the development of LMN signs on examination, though some have reported a latency exceeding 4 years.^3^

Singer *et al* divided patients into two categories, with and without EMG findings.^20^ Neither group went on to develop ALS. They found the group with ‘evidence of active denervation potentials (increased insertional activity, fibrillations and/or positive sharp waves) in one or more muscles’ were older and progressed more rapidly. None of the other series reported any significance to minor EMG findings. Mitsumoto *et al* included patients with normal EMG but allowed minimal changes in one muscle.^21^ Fournier *et al* identified 217 patients with pure UMN disease at 20 clinic sites and divided the groups into normal EMG, and minor denervation, and found no differences between groups.^22^

In general, most patients with minor denervation in a rare extremity muscle remain a pure UMN syndrome, so that these minimal findings on EMG have been permitted. The inverse, someone with minor EMG findings in a rare muscle initially who at 4 years have a completely normal EMG, would also support a PLS diagnosis. Notwithstanding the limitations of using EMG as a biomarker for LMN involvement, the recognition of a pragmatic category of ‘probable PLS’ for those with a progressive, idiopathic upper motor syndrome of between 2 and 4 years from symptom onset, reflects a desire to facilitate earlier inclusion of patients with PLS in future trials of potentially disease-modifying therapy before disability becomes advanced.

### Differential diagnosis

HSP has the most clinical overlap with the early symptomatic phase of lower limb-onset PLS.^24^ HSP and PLS are both fundamentally *clinical* syndromes. A significant proportion of individuals confidently labelled as HSP on clinical grounds will not carry a recognised pathological genetic variant. Analysis of 90 patients with apparently sporadic UMN syndrome with phenotypes of HSP (involvement of legs only), HSP-PLS overlap (involvement of arms and legs) and PLS (bulbar involvement) showed significant overlap in the age of symptom onset and no differences between the groups in features classically used to distinguish the two, such as mild dorsal column dysfunction or urinary urgency.^25^

Mimic disorders for PLS are rare and high-resolution clinical MRI of the brain and spinal cord will eliminate the majority of these. With the exception of the neurodegenerative category, the plausibility of many alternative diagnoses greatly diminishes with the duration of a progressive pure UMN syndrome at the time of clinical assessment (table 1).

**Table 1:** Differential diagnosis of primary lateral sclerosis

Neurodegenerative	Key distinguishing features
Upper motor neuron-predominant amyotrophic lateral sclerosis Hereditary spastic paraparesis Alexander disease	Development of clinically progressive lower motor neuron involvement. Family history or relevant genetic variant; symmetrical weakness limited to lower limbs. Focal atrophy and MRI signal change in the medulla, or pathogenic variant in *GFAP.*
**Neuroinflammatory**	
Primary progressive multiple sclerosis Anti-amphiphysin paraneoplastic syndrome	Inflammatory lesions on MRI of the brain and cord. Positive antibody in context of coincident malignancy.
**Metabolic**	
Adrenomyeloneuropathy	Cerebral MRI white matter abnormalities; raised serum very long chain fatty acids; pathogenic variant in *ABCD1.*
**Infectious**	
Tropical spastic paraparesis (Human T-cell lymphotropic virus, HTLV-1 & 2) Syphilis	Positive IgM serology. Positive serology.
**Structural**	
Foramen magnum region lesions Parafalcine meningioma	MRI appearances. MRI appearances.
**Vascular**	
Spinal arteriovenous malformation	MRI appearances.

It is not a requirement that all are formally excluded, rather investigations are guided by clinical plausibility.

### The role of genetic testing

The classical syndrome of PLS appears to be sporadic and the diagnosis based on clinical features. Screening of panels for pathogenic genetic variants associated with spastic paraparesis (eg, *SPAST*) is warranted in cases of progressive UMN syndromes restricted to symmetrical lower limb involvement. It is reasonable to routinely exclude the most common hereditary cause of ALS in Caucasian populations, namely an expansion in *C9orf72* which may present with an UMN-predominant phenotype. However, the plethora of very rare genetic variants reported in association with pedigrees containing ALS-like syndromes, including some with apparently pure UMN phenotypes, should *not* be considered routine tests in the diagnosis of PLS (box 2).

Box 2 **Genetic variants reported in a small percentage of upper motor neuron-predominant syndromes**

SPG7*

ALS2*

D4S2963*

C9orf72

DCTN1

PARK2

ERLIN2

FIG4

SYNE2

VEGFA

CLN6

BTD

LRKK2

SQSTM1*

KIF5a*

KIF1a

Primary lateral sclerosis appears to be a sporadic disorder essentially, with diagnosis based primarily on clinical features rather than genotype.*Familial cases.

### Emerging technology

A range of neuroimaging and neurophysiological tools have clear potential to quantify the UMN lesion and may help to refine the diagnostic pathway for PLS.^26^ Conversely, tools aimed at demonstrating subclinical LMN involvement, such as muscle ultrasound for the detection of fasciculation,^27–29^ may ultimately offer additional value for the earlier distinction from UMN-predominant ALS.

Transcranial magnetic stimulation studies have noted greater central motor conduction times in PLS compared with ALS,^18^ in addition to high threshold measures for cortical stimulation, leading to the development of relative cortical inexcitability, a feature that reliably distinguishes PLS from HSP.^30^ Focus on beta-band EMG has considered intermuscular coherence as a potential distinguisher of PLS from ALS,^31^ and magnetoencephalography offers a broader analysis of differences in corticomuscular coherence.^32^

Neurofilaments are an emerging biofluid biomarker reflecting the intensity of neuronal loss in a range of neurological disorders. Levels tend to be much lower in PLS compared with ALS,^33^ reflecting its much slower progression. Cerebrospinal fluid chitinases, thought to be macrophage-derived, may show a differential pattern of involvement in PLS compared with ALS,^34^ but further studies are needed to explore the key distinction of PLS from UMN-predominant ALS.

**Figure 1:**
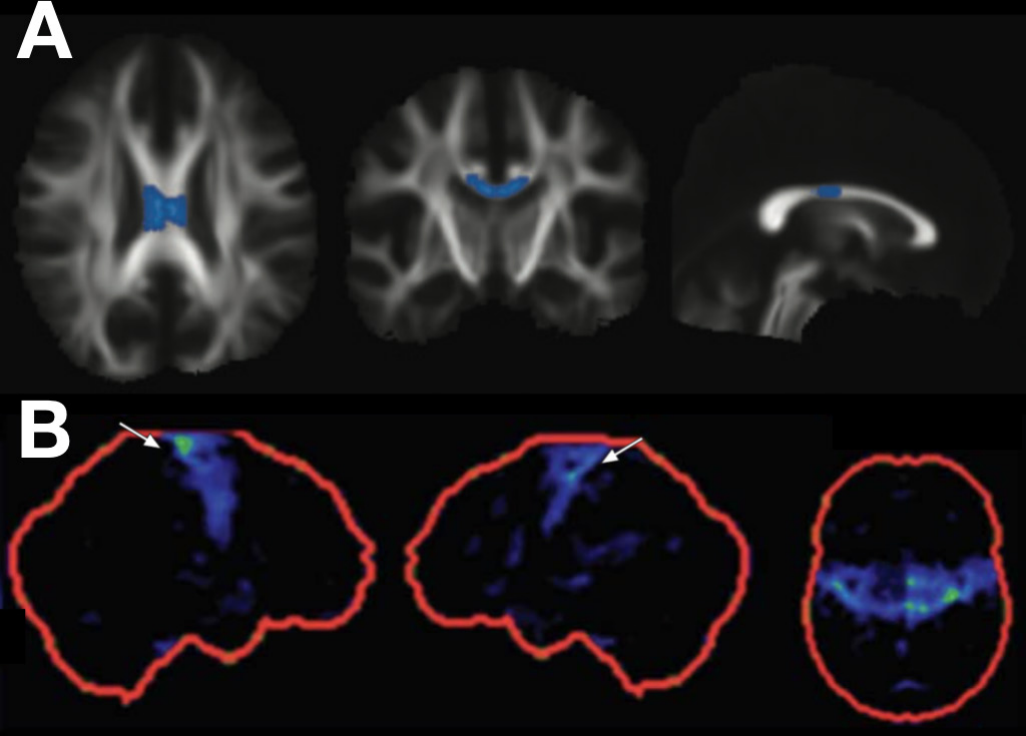
Diffusion tensor imaging and fluorodeoxyglucose (FDG) positron emission tomography (PET) findings in cases of primary lateral sclerosis (PLS). Mean diffusivity in the mid-portion of the corpus callosum is increased in cases of PLS compared with amyotrophic lateral sclerosis (row A, PLS vs ALS group findings highlighted on axial, coronal and sagittal views of the white matter tract skeleton; with kind permission of the author, see Iwata *et al* 37). Focal hypometabolism may be seen in the primary motor cortices in PLS (row B, FDG PET z-score images in the right sagittal, left sagittal and axial planes; with kind permission of the author, see Claassen *et al* 35). Neither of these techniques yet has sufficient sensitivity or specificity to be applied in isolation for the diagnosis of PLS.

One of the features noted in some established cases of PLS is a focal ‘knife edge’ atrophy of the precentral gyrus,^4^ which is strikingly absent in even advanced cases of ALS. With the refinement of more automated volumetric MRI analysis, coupled to large normative databases, it may be possible to quantify the degree of focal motor cortical atrophy at the individual patient level and integrate this within the diagnostic certainty algorithm. Similarly, the presence of focal fluorodeoxyglucose hypometabolism in the same region—the ‘stripe sign’—has been associated with PLS,^35^ but has not been validated against the core differential diagnosis of UMN-predominant ALS. Diffusion tensor imaging is a development of standard MRI permitting assessment of the integrity of large white matter tracts through the surrogate marker of the directionality of water diffusion. This suggests greater white matter damage in the region of the central corpus callosum in patients with PLS, but this currently lacks the sensitivity and specificity for the diagnosis of individual patients^36–38^ (figure 1). Cerebellar involvement,^38 39^ corticospinal tract fluid attenuated inversion recovery (FLAIR) hyperintensity^40^ and quantitative susceptibility mapping of iron deposition in the motor cortex^41^ have all been noted as increased in PLS. The continued development of volumetric spinal cord imaging and its integration with cerebral structural measures may offer greater potential for distinguishing PLS cases.^42^

All of these tools require further prospective studies to define their precise value in the diagnostic algorithm for PLS.

## Conclusions

Developments in neuroimaging, neurophysiology and molecular biology have not diminished the long-standing recognition of PLS as a distinct, *clinical* *ly*-defined syndrome. Its rarity and prolonged survival trajectory have resulted in a degree of neglect in terms of therapeutic trials compared with ALS. Traditional reliance on EMG with overinterpretation of minimal markers of LMN involvement may contribute to significant diagnostic delay. The development of an international registry that includes all those with ‘probable PLS’ will allow a more precise delineation of the pathogenesis from ALS, and accelerate therapeutic developments.

Beyond primary disease-modifying therapy, the most pressing unmet need for patients with PLS may well be the treatment of core symptoms rather than extension of survival. The development of more effective relief of spasticity that does not sacrifice muscle strength would have a great impact for those living with PLS, notwithstanding the long-term desire for neuroprotective or regenerative therapy. Recognising that advances in molecular phenotyping may well supersede purely clinical diagnostics, it is hoped that these pragmatic criteria will provide greater confidence to reduce diagnostic delay, thus allowing access to potentially disease-modifying therapies at lower levels of disability.

## References

[1] Stark FM, Moersch FP Primary lateral sclerosis: a distinct clinical entity. J Nerv Ment Dis 1945;102:332–7.

[2] Chiò A, Calvo A, Moglia C, et al Phenotypic heterogeneity of amyotrophic lateral sclerosis: a population based study. J Neurol Neurosurg Psychiatry 2011;82:740–6. 10.1136/jnnp.2010.235952 21402743

[3] D'Amico E, Pasmantier M, Lee Y-W, et al Clinical evolution of pure upper motor neuron disease/dysfunction (PUMMD). Muscle Nerve 2013;47:28–32. 10.1002/mus.23496 23169452 PMC3528840

[4] Pringle CE, Hudson AJ, Munoz DG, et al Primary lateral sclerosis. clinical features, neuropathology and diagnostic criteria. Brain 1992;115:495–520. 10.1093/brain/115.2.495 1606479

[5] Gordon PH, Cheng B, Katz IB, et al The natural history of primary lateral sclerosis. Neurology 2006;66:647–53. 10.1212/01.wnl.0000200962.94777.71 16534101

[6] Tan C-F, Kakita A, Piao Y-S, et al Primary lateral sclerosis: a rare upper-motor-predominant form of amyotrophic lateral sclerosis often accompanied by frontotemporal lobar degeneration with ubiquitinated neuronal inclusions? report of an autopsy case and a review of the literature. Acta Neuropathol 2003;105:615–20. 10.1007/s00401-003-0687-0 12734667

[7] Dickson DW, Josephs KA, Amador-Ortiz C Tdp-43 in differential diagnosis of motor neuron disorders. Acta Neuropathol 2007;114:71–9. 10.1007/s00401-007-0234-5 17569066

[8] Singer MA, Statland JM, Wolfe GI, et al Primary lateral sclerosis. Muscle Nerve 2007;35:291–302. 10.1002/mus.20728 17212349

[9] Zhai P, Pagan F, Statland J, et al Primary lateral sclerosis: a heterogeneous disorder composed of different subtypes? Neurology 2003;60:1258–65. 10.1212/01.WNL.0000058900.02672.D2 12707427

[10] Norlinah IM, Bhatia KP, Østergaard K, et al Primary lateral sclerosis mimicking atypical parkinsonism. Mov Disord 2007;22:2057–62. 10.1002/mds.21645 17702034

[11] Mabuchi N, Watanabe H, Atsuta N, et al Primary lateral sclerosis presenting parkinsonian symptoms without nigrostriatal involvement. J Neurol Neurosurg Psychiatry 2004;75:1768–71. 10.1136/jnnp.2003.035212 15548503 PMC1738849

[12] de Vries BS, Rustemeijer LMM, Bakker LA, et al Cognitive and behavioural changes in PLS and PMA:challenging the concept of restricted phenotypes. J Neurol Neurosurg Psychiatry 2019;90:141–7. 10.1136/jnnp-2018-318788 30076267

[13] Agarwal S, Highton-Williamson E, Caga J, et al Primary lateral sclerosis and the amyotrophic lateral sclerosis–frontotemporal dementia spectrum. J Neurol 2018;265:1819–28. 10.1007/s00415-018-8917-5 29868980

[14] Turner MR The reunification of amyotrophic lateral sclerosis. J Neurol Neurosurg Psychiatry 2019;90:122–3. 10.1136/jnnp-2018-319470 30297521

[15] Mills CK Unilateral ascending paralysis and unilateral descending paralysis. their clinical varieties and their pathological causes. JAMA 1906;20:1638–45.

[16] Keegan BM, Kaufmann TJ, Weinshenker BG, et al Progressive solitary sclerosis: gradual motor impairment from a single CNS demyelinating lesion. Neurology 2016;87:1713–9. 10.1212/WNL.0000000000003235 27638926 PMC5085075

[17] Gastaut JL, Bartolomei F Mills' syndrome: ascending (or descending) progressive hemiplegia: a hemiplegic form of primary lateral sclerosis? J Neurol Neurosurg Psychiatry 1994;57:1280–1. 10.1136/jnnp.57.10.1280 7931406 PMC485513

[18] Kuipers-Upmeijer J, de Jager AE, Hew JM Primary lateral sclerosis: clinical, neurophysiological, and magnetic resonance findings. JNeurolNeurosurgPsychiatry 2001;71:615–20. 10.1136/jnnp.71.5.615 PMC1737610 11606672

[19] Le Forestier N, Maisonobe T, Piquard A, et al Does primary lateral sclerosis exist? A study of 20 patients and a review of the literature. Brain 2001;124:1989–99. 10.1093/brain/124.10.1989 11571217

[20] Singer MA, Kojan S, Barohn RJ, et al Primary lateral sclerosis: clinical and laboratory features in 25 patients. J Clin Neuromuscul Dis 2005;7:1–9. 10.1097/01.cnd.0000176974.61136.45 19078775

[21] Mitsumoto H, Nagy PL, Gennings C, et al Phenotypic and molecular analyses of primary lateral sclerosis. Neurol Genet 2015;1:e3 10.1212/01.NXG.0000464294.88607.dd 27066542 PMC4821084

[22] Fournier CN, Murphy A, Loci L, et al Primary lateral sclerosis and early upper motor neuron disease: characteristics of a cross-sectional population. J Clin Neuromuscul Dis 2016;17:99–105. 10.1097/CND.0000000000000102 26905909 PMC4770823

[23] Wais V, Rosenbohm A, Petri S, et al The concept and diagnostic criteria of primary lateral sclerosis. Acta Neurol Scand 2017;136:204–11. 10.1111/ane.12713 27858953

[24] Fink JK Progressive spastic paraparesis: hereditary spastic paraplegia and its relation to primary and amyotrophic lateral sclerosis. Semin Neurol 2001;21:199–208. 10.1055/s-2001-15265 11442328

[25] Brugman F, Veldink JH, Franssen H, et al Differentiation of hereditary spastic paraparesis from primary lateral sclerosis in sporadic adult-onset upper motor neuron syndromes. Arch Neurol 2009;66:509–14. 10.1001/archneurol.2009.19 19364936

[26] Huynh W, Simon NG, Grosskreutz J, et al Assessment of the upper motor neuron in amyotrophic lateral sclerosis. Clinical Neurophysiology 2016;127:2643–60. 10.1016/j.clinph.2016.04.025 27291884

[27] Tsuji Y, Noto Y-ichi, Shiga K, et al A muscle ultrasound score in the diagnosis of amyotrophic lateral sclerosis. Clinical Neurophysiology 2017;128:1069–74. 10.1016/j.clinph.2017.02.015 28343888

[28] Noto Y-I, Shibuya K, Shahrizaila N, et al Detection of fasciculations in amyotrophic lateral sclerosis: the optimal ultrasound scan time. Muscle Nerve 2017;56:1068–71. 10.1002/mus.25607 28187527

[29] Tsugawa J, Dharmadasa T, Ma Y, et al Fasciculation intensity and disease progression in amyotrophic lateral sclerosis. Clin Neurophysiol 2018;129:2149–54. 10.1016/j.clinph.2018.07.015 30114663

[30] Geevasinga N, Menon P, Sue CM, et al Cortical excitability changes distinguish the motor neuron disease phenotypes from hereditary spastic paraplegia. Eur J Neurol 2015;22:826–58. 10.1111/ene.12669 25683471

[31] Fisher KM, Zaaimi B, Williams TL, et al Beta-Band intermuscular coherence: a novel biomarker of upper motor neuron dysfunction in motor neuron disease. Brain 2012;135:2849–64. 10.1093/brain/aws150 22734124 PMC3437020

[32] Proudfoot M, van Ede F, Quinn A, et al Impaired corticomuscular and interhemispheric cortical beta oscillation coupling in amyotrophic lateral sclerosis. Clin Neurophysiol 2018;129:1479–89. 10.1016/j.clinph.2018.03.019 29678369

[33] Steinacker P, Feneberg E, Weishaupt J, et al Neurofilaments in the diagnosis of motoneuron diseases: a prospective study on 455 patients. J Neurol Neurosurg Psychiatry 2016;87:12–20. 10.1136/jnnp-2015-311387 26296871

[34] Thompson AG, Gray E, Bampton A, et al Csf chitinase proteins in amyotrophic lateral sclerosis. J Neurol Neurosurg Psychiatry 2019;90:1215–20. 10.1136/jnnp-2019-320442 31123140

[35] Claassen DO, Josephs KA, Peller PJ The stripe of primary lateral sclerosis: focal primary motor cortex hypometabolism seen on fluorodeoxyglucose F18 positron emission tomography. Arch Neurol 2010;67:122–5. 10.1001/archneurol.2009.298 20065142

[36] Ciccarelli O, Behrens TE, Johansen-Berg H, et al Investigation of white matter pathology in ALS and PLS using tract-based spatial statistics. Hum Brain Mapp 2009;30:615–24. 10.1002/hbm.20527 18172851 PMC6870826

[37] Iwata NK, Kwan JY, Danielian LE, et al White matter alterations differ in primary lateral sclerosis and amyotrophic lateral sclerosis. Brain 2011;134:2642–55. 10.1093/brain/awr178 21798965 PMC3170531

[38] Finegan E, Chipika RH, Li Hi Shing S, et al The clinical and radiological profile of primary lateral sclerosis: a population-based study. J Neurol 2019;266:2718–33. 10.1007/s00415-019-09473-z 31325016

[39] Tu S, Menke RAL, Talbot K, et al Cerebellar tract alterations in PLS and ALS. Amyotroph Lateral Scler Frontotemporal Degener 2019;20:281–4. 10.1080/21678421.2018.1562554 30663900

[40] Fabes J, Matthews L, Filippini N, et al Quantitative FLAIR MRI in amyotrophic lateral sclerosis. Acad Radiol 2017;24:1187–94. 10.1016/j.acra.2017.04.008 28572001 PMC5605225

[41] Schweitzer AD, Liu T, Gupta A, et al Quantitative susceptibility mapping of the motor cortex in amyotrophic lateral sclerosis and primary lateral sclerosis. AJR Am J Roentgenol 2015;204:1086–92. 10.2214/AJR.14.13459 25905946 PMC4889122

[42] van der Burgh HK, Westeneng H-J, Meier JM, et al Cross-Sectional and longitudinal assessment of the upper cervical spinal cord in motor neuron disease. Neuroimage 2019;24:101984 10.1016/j.nicl.2019.101984 31499409 PMC6734179

