# Correction: *Primary lateral sclerosis: consensus diagnostic criteria*

**DOI:** 10.1136/jnnp-2019-322541corr1

**Published:** 2020-07-17

Turner MR, Barohn RJ, Corcia P, *et al*. Primary lateral sclerosis: consensus diagnostic criteria. *J Neurol Neurosurg Psychiatry* 2020;91:373–7.

In this paper, Georg Haase should have been included in the collaborators list.

